# Longitudinal study of the inverse relationship between Parkinson’s disease and cancer in Korea

**DOI:** 10.1038/s41531-023-00562-5

**Published:** 2023-07-22

**Authors:** So Young Kim, Hyo Geun Choi, Yoo Hwan Kim, Mi Jung Kwon, Joo-Hee Kim, Heui Seung Lee, Ji Hee Kim

**Affiliations:** 1 Department of Otorhinolaryngology-Head & Neck Surgery, CHA Bundang Medical Center, CHA University, Seongnam, Korea; 2 MD Analytics, Seoul, Korea; 3 Suseoseoul ENT Clinic, Department of Otorhinolaryngology-Head & Neck Surgery, Seoul, Korea; 4 Department of Neurology, Hallym University College of Medicine, Anyang, Korea; 5 Department of Pathology, Hallym University College of Medicine, Anyang, Korea; 6 Division of Pulmonary, Allergy, and Critical Care Medicine, Department of Medicine, Hallym University College of Medicine, Anyang, Korea; 7 Department of Neurosurgery, Hallym University College of Medicine, Anyang, Korea

**Keywords:** Cancer, Neurological disorders, Parkinson's disease

## Abstract

Despite growing epidemiological evidence, the relationship between Parkinson’s disease (PD) and cancer has not been conclusively demonstrated, and related studies are scarce in the Asian population. We aimed to determine the association between PD and subsequent development of various cancers from longitudinal data of a representative sample of Korean adults aged ≥40 years. We retrospectively identified 8381 patients diagnosed with PD from 2002 to 2019 using claims data among 514,866 people of random samples from the Korean National Health Insurance database. We sampled 33,524 age-, sex-, income-, and residential area-matched participants without PD from the same database. The longitudinal associations between PD and overall cancer, as well as 10 common types of cancer, were estimated using multivariable Cox proportional-hazards regression analysis. The adjusted hazard ratio (aHR) of all cancer types was 0.63 (95% confidence interval = 0.57–0.69) in patients with PD compared with matched controls. The aHRs of gastric, thyroid, colorectal, lung, hepatic, and pancreatic cancer and hematological malignancy were 0.69 (0.56–0.85), 0.60 (0.39–0.93), 0.56 (0.44–0.70), 0.71 (0.58–0.84), 0.64 (0.48–0.86), 0.37 (0.23–0.60), and 0.56 (0.36–0.87), respectively. The associations of bladder, gallbladder and biliary duct, and kidney cancer with PD were not statistically significant. Our findings show inverse associations between overall cancer and most cancer types in patients with PD. These inverse associations and their pathogeneses merit further investigation.

## Introduction

Parkinson’s disease (PD) is the second most prevalent degenerative disease of the central nervous system (CNS), affecting approximately 1–2% of the world population. PD is characterized by an imbalance in the output of the striatum owing to progressive loss of nigrostriatal dopaminergic innervation, resulting in cardinal motor symptoms, such as bradykinesia, 4–6 Hz resting tremor, rigidity, and postural instability, as well as autonomic dysfunction, psychological changes, and cognitive impairment. Although the exact pathogenesis of PD is still not understood, it is considered a multifactorial disease caused by the interaction of genetic, environmental, and endogenous factors.

The characteristic pathological outcomes of PD are extensive neurodegenerative changes and cell death throughout the nervous system, whereas cancer pathology is based on an increased cell proliferation and resistance to cell death due to uncontrolled cell division. Therefore, from cell cycle perspective, these two diseases are on opposite ends of a spectrum. Although these two disease categories are seemingly unrelated, neurodegeneration and cancer have common molecular properties, such as overlapping genetic and biochemical pathways, implying that genetic background that is protective against cancer may also predispose an individual to neurodegeneration, such as in PD, and vice versa^1,2^. Numerous epidemiological observations also bear out this phenomenon: patients with PD have a lower risk of developing many common types of cancer than do the general population^3–5^. However, these epidemiological reports were mostly from Western countries. To our knowledge, only three studies of overall cancer development in Asian patients with PD have been reported, and their results were inconsistent^6–8^.

Insight into the connections between these two distinct disorders may elucidate their pathogeneses. As differences in genetic background or susceptibility across ethnicities may modify the pathogeneses of both PD and cancer, more data in Asian populations sharing similar genetic properties are needed to determine the epidemiological relationship between the two diseases.

Hence, in this study, we aimed to determine the association between PD and the development of cancer, as well as 10 common types of cancer in particular, in the Korean adult population.

## Results

### Baseline characteristics of the study population

**Fig. 1:**
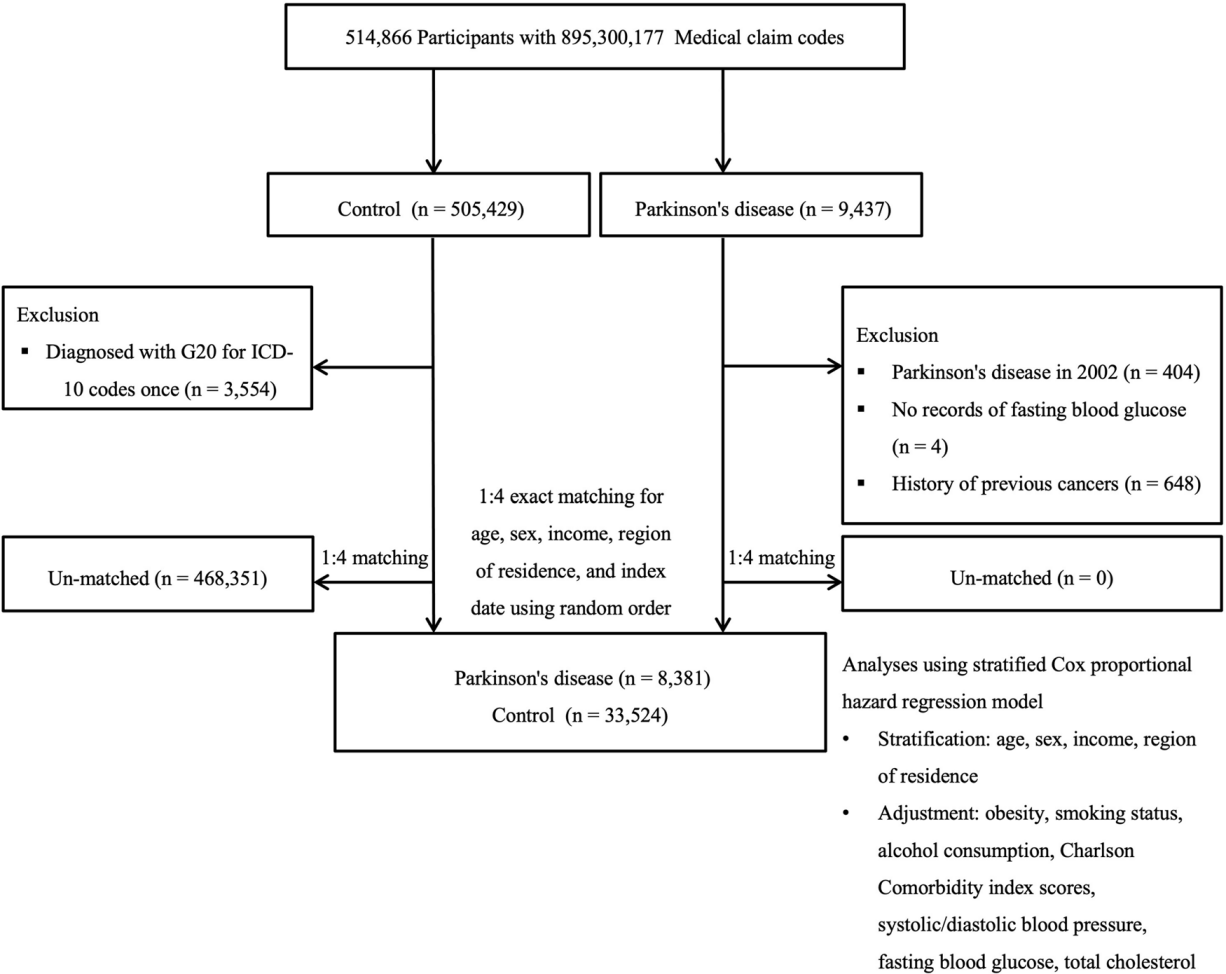
A schematic representation of the participant selection process used in this study. Ultimately, 8381 participants with PD were matched with 33,524 control participants based on age, sex, income, and region of residence out of a total of 514,866 participants in the database.

After propensity score matching, a total of 8381 patients with PD and 33,524 matched controls were included in the analyses (Fig. 1). In the PD group, 46.73% and 77.61% of patients were men and nonsmokers, respectively, and 71.66% of them consumed alcohol <1 time a week. Most of the baseline characteristics were similar for patients with PD and their controls (except for the CCI [Charlson Comorbidity Index] score, standardized difference = 0.39) before applying overlap weighting. After weighting, satisfactory across-group balances were achieved (all standardized differences = 0.00, Table 1).

**Table 1:** General characteristics of participants.

Characteristics	Before overlap weighting adjustment			After overlap weighting adjustment		
	Parkinson’s disease	Control	Standardized difference	Parkinson’s disease	Control	Standardized difference
Age (*n*, %)			0.00			0.00
40–44	8 (0.10)	32 (0.10)		6 (0.09)	6 (0.09)	
45–49	72 (0.86)	288 (0.86)		57 (0.86)	57 (0.86)	
50–54	233 (2.78)	932 (2.78)		183 (2.78)	183 (2.78)	
55–59	496 (5.92)	1984 (5.92)		389 (5.91)	389 (5.91)	
60–64	874 (10.43)	3496 (10.43)		683 (10.39)	683 (10.39)	
65–69	1298 (15.49)	5192 (15.49)		1017 (15.47)	1017 (15.47)	
70–74	1868 (22.29)	7472 (22.29)		1460 (22.20)	1460 (22.20)	
75–79	1975 (23.57)	7900 (23.57)		1552 (23.60)	1552 (23.60)	
80–84	1185 (14.14)	4740 (14.14)		935 (14.22)	935 (14.22)	
85+	372 (4.44)	1488 (4.44)		295 (4.48)	295 (4.48)	
Sex (*n*, %)			0.00			0.00
Men	3919 (46.76)	15,676 (46.76)		3073 (46.73)	3073 (46.73)	
Women	4462 (53.24)	17,848 (53.24)		3503 (53.27)	3503 (53.27)	
Income (*n*, %)			0.00			0.00
1 (lowest)	1541 (18.39)	6164 (18.39)		1205 (18.32)	1205 (18.32)	
2	917 (10.94)	3668 (10.94)		719 (10.94)	719 (10.94)	
3	1135 (13.54)	4540 (13.54)		891 (13.55)	891 (13.55)	
4	1634 (19.50)	6536 (19.50)		1283 (19.51)	1283 (19.51)	
5 (highest)	3154 (37.63)	12,616 (37.63)		2478 (37.67)	2478 (37.67)	
Region of residence (*n*, %)			0.00			0.00
Urban	3141 (37.48)	12,564 (37.48)		2462 (37.44)	2462 (37.44)	
Rural	5240 (62.52)	20,960 (62.52)		4114 (62.56)	4114 (62.56)	
Obesity^a^ (*n*, %)		0.01			0.00	
Underweight	306 (3.65)	1205 (3.59)		239 (3.63)	239 (3.63)	
Normal	2931 (34.97)	11,795 (35.18)		2302 (35.00)	2302 (35.00)	
Overweight	2206 (26.32)	8803 (26.26)		1734 (26.37)	1734 (26.37)	
Obese I	2654 (31.67)	10,615 (31.66)		2080 (31.64)	2080 (31.64)	
Obese II	284 (3.39)	1106 (3.30)		221 (3.36)	221 (3.36)	
Smoking status (*n*, %)			0.09			0.00
Nonsmoker	6566 (78.34)	24,986 (74.53)		5104 (77.61)	5104 (77.61)	
Past smoker	1060 (12.65)	4633 (13.82)		850 (12.92)	850 (12.92)	
Current smoker	755 (9.01)	3905 (11.65)		623 (9.47)	623 (9.47)	
Alcohol consumption (*n*, %)			0.11			0.00
<1 time a week	6089 (72.65)	22,723 (67.78)		4712 (71.66)	4712 (71.66)	
≥1 time a week	2292 (27.35)	10,801 (32.22)		1864 (28.34)	1864 (28.34)	
Systolic blood pressure (Mean, SD)	130.00 (17.49)	130.23 (17.05)	0.02	130.04 (15.48)	130.04 (7.56)	0.00
Diastolic blood pressure (Mean, SD)	78.43 (10.79)	78.35 (10.54)	0.02	78.41 (9.55)	78.41 (4.70)	0.00
Fasting blood glucose (Mean, SD)	106.93 (36.51)	103.83 (30.89)	0.09	106.03 (30.46)	106.03 (16.18)	0.00
Total cholesterol (Mean, SD)	194.61 (43.93)	196.41 (39.45)	0.05	195.01 (39.52)	195.01 (17.47)	0.00
CCI score (Mean, SD)	1.76 (1.90)	1.21 (1.75)	0.39	1.63 (1.59)	1.63 (0.94)	0.00
Overall cancer (*n*, %)	469 (5.60)	2696 (8.04)	0.1	334 (5.07)	729 (11.08)	0.22
Gastric cancer (*n*, %)	112 (1.34)	577 (1.72)	0.03	81 (1.23)	154 (2.34)	0.08
Thyroid cancer (*n*, %)	24 (0.29)	136 (0.41)	0.02	18 (0.27)	36 (0.54)	0.04
Colorectal cancer (*n*, %)	83 (0.99)	554 (1.65)	0.06	59 (0.90)	147 (2.24)	0.11
Lung cancer (*n*, %)	114 (1.36)	642 (1.92)	0.04	80 (1.22)	174 (2.64)	0.1
Hepatic cancer (*n*, %)	53 (0.63)	311 (0.93)	0.03	36 (0.55)	93 (1.41)	0.09
Bladder cancer (*n*, %)	29 (0.35)	165 (0.49)	0.02	21 (0.32)	41 (0.62)	0.04
Pancreatic cancer (*n*, %)	19 (0.23)	182 (0.54)	0.05	14 (0.21)	54 (0.82)	0.09
Gallbladder and biliary duct cancer (*n*, %)	37 (0.44)	164 (0.49)	0.01	26 (0.39)	47 (0.72)	0.04
Kidney cancer (*n*, %)	22 (0.26)	70 (0.21)	0.01	16 (0.24)	19 (0.29)	0.01
Hematological malignancy (*n*, %)	23 (0.27)	152 (0.45)	0.03	17 (0.26)	39 (0.59)	0.05

*CCI* Charlson comorbidity index, *SD* standard deviation.

^a^Obesity (BMI, body mass index, kg/m^2^) was categorized as <18.5 (underweight), ≥18.5 to <23 (normal), ≥23 to <25 (overweight), ≥25 to <30 (obese I), and ≥30 (obese II).

### Incidence rates and hazard ratios of cancer in patients with PD

The incidence rates of overall cancer were 11.52 and 14.09 per 1000 person years, respectively, for the PD and control groups (Table 2). During the follow-up period, the adjusted hazard ratio (aHR) for overall cancer in model 2 was 0.63 (95% CI = 0.57–0.69) in patients with PD compared with matched controls. Likewise, the aHRs for gastric, thyroid, colorectal, lung, hepatic, and pancreatic cancer and hematological malignancy during the follow-up period were 0.69 (95% CI [confidence interval] = 0.56–0.85), 0.60 (95% CI = 0.39–0.93), 0.56 (95% CI = 0.44–0.70), 0.71 (95% CI = 0.58–0.84), 0.64 (95% CI = 0.48–0.86), 0.37 (95% CI = 0.23–0.60), and 0.56 (95% CI = 0.36–0.87). Compared with control participants, those with PD had lower HRs of subsequent bladder cancer (aHR = 0.71, 95% CI = 0.48–1.06) and gallbladder and biliary duct cancer (aHR = 0.87, 95% CI = 0.61–1.26) and a higher HR of developing kidney cancer (aHR = 1.14, 95% CI = 0.70–1.86), but these differences were not statistically significant (Table 2).

**Table 2:** Crude and adjusted hazard ratios of Parkinson’s disease for various cancers.

	Incident rate per 1000 person-year		Incident rate difference per 1000 person-years (95% CI)	Hazard ratio for cancer (95% CI)					
	PD	Control		Crude^b^	*P*-value	Model 1^b,c^	*P*-value	Model 2^b,c,d^	*P*-value
Overall cancer (*n* = 3165)	11.52	14.09	−2.57 (−3.82 to −1.32)	0.82 (0.74–0.91)	<0.001^a^	0.62 (0.56–0.68)	<0.001^a^	0.63 (0.57–0.69)	<0.001^a^
Gastric cancer (*n* = 689)	2.70	2.95	−0.25 (−0.82 to 0.32)	0.91 (0.75–1.12)	0.387	0.68 (0.56–0.84)	<0.001^a^	0.69 (0.56–0.85)	<0.001^a^
Thyroid cancer (*n* = 160)	0.57	0.69	−0.11 (−0.39 to 0.16)	0.76 (0.49–1.17)	0.216	0.58 (0.38–0.90)	0.015^a^	0.60 (0.39–0.93)	0.022^a^
Colorectal cancer (*n* = 637)	1.98	2.81	−0.83 (−1.37 to −0.28)	0.71 (0.56–0.89)	0.004^a^	0.55 (0.44–0.70)	<0.001^a^	0.56 (0.44–0.70)	<0.001^a^
Lung cancer (*n* = 756)	2.71	3.24	−0.53 (−1.12 to 0.06)	0.86 (0.71–1.06)	0.153	0.69 (0.57–0.85)	<0.001^a^	0.71 (0.58–0.87)	0.001^a^
Hepatic cancer (*n* = 364)	1.26	1.57	−0.31 (−0.72 to 0.10)	0.83 (0.62–1.11)	0.197	0.64 (0.47–0.86)	0.003^a^	0.64 (0.48–0.86)	0.003^a^
Bladder cancer (*n* = 194)	0.69	0.83	−0.14 (−0.44 to 0.16)	0.86 (0.58–1.28)	0.462	0.71 (0.48–1.06)	0.091	0.71 (0.48–1.06)	0.091
Pancreatic cancer (*n* = 201)	0.45	0.91	−0.46 (−0.77 to −0.16)	0.50 (0.31–0.80)	0.004^a^	0.36 (0.22–0.58)	0.001^a^	0.37 (0.23–0.60)	0.001^a^
Gallbladder and biliary duct cancer (*n* = 201)	0.88	0.82	0.05 (−0.25 to 0.36)	1.07 (0.75–1.52)	0.730	0.85 (0.59–1.23)	0.389	0.87 (0.61–1.26)	0.461
Kidney cancer (*n* = 92)	0.52	0.35	0.17 (−0.03 to 0.38)	1.41 (0.87–2.28)	0.161	1.14 (0.70–1.85)	0.606	1.14 (0.70–1.86)	0.597
Hematological malignancy (*n* = 175)	0.55	0.76	−0.22 (−0.50 to 0.06)	0.70 (0.45–1.08)	0.109	0.56 (0.36–0.88)	0.011^a^	0.56 (0.36–0.87)	0.010^a^

*CI* confidence interval, *PD* Parkinson’s disease.

^a^Stratified Cox proportional hazard regression model, Significance at *P* < 0.05.

^b^Stratified by age, sex, income, and region of residence.

^c^Adjusted for obesity, smoking, alcohol consumption, and Charlson Comorbidity Index scores.

^d^Adjusted for systolic blood pressure, diastolic blood pressure, fasting blood glucose, and total cholesterol.

**Fig. 2:**
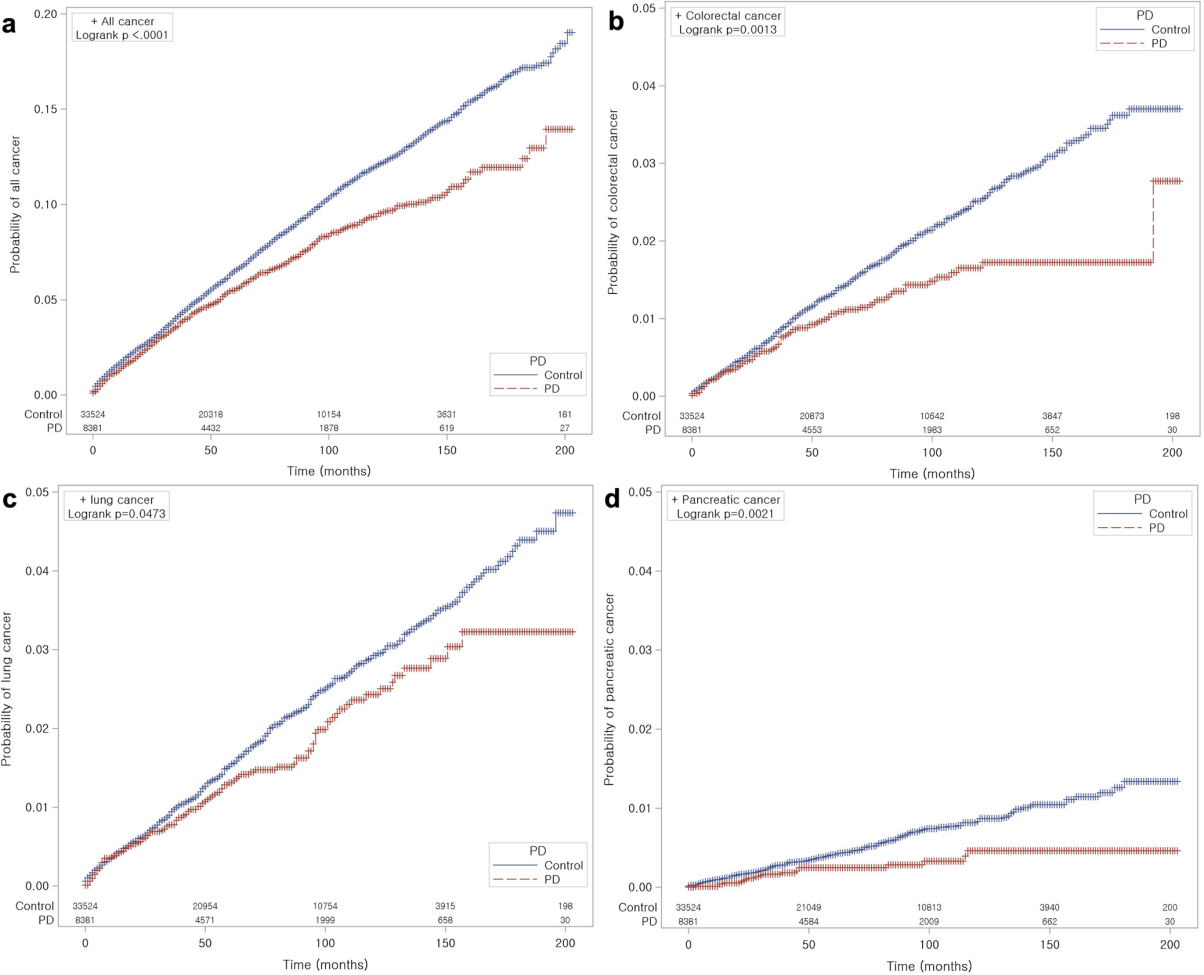
Kaplan–Meier curves and statistical comparisons of the time to development of cancer in PD and control groups. The incidence probabilities of overall cancer (**a**), colorectal cancer (**b**), lung cancer (**c**), and pancreatic cancer (**d**) were lower in patients with PD than in controls.

Figure 2 contains the survival curves of patients with PD versus controls for all cancer types together and per cancer type. The incidence probabilities of overall, colorectal, lung, and pancreatic cancer in patients with PD were significantly lower than those of the control group (each *P* < 0.05, log-rank test).

### Subgroup analysis

Sex-stratified analysis revealed aHRs of 0.66 (95% CI = 0.58–0.75) and 0.56 (95% CI = 0.48–0.66), respectively, for the development of any cancer in men and women with PD. The significant inverse associations of PD with gastric, colorectal, and pancreatic cancer were consistent across both subgroups. In addition, while PD was inversely associated with the development of lung and hepatic cancer among men, PD in the subgroup of women was inversely linked to subsequent hematological malignancy (Table 3).

**Table 3:** Sex-stratified subgroup analysis of crude and adjusted hazard ratios of Parkinson’s disease for various cancers.

	Incident rate per 1000 person-year		Incident rate difference per 1000 person-years (95% CI)	Hazard ratio for cancer (95% CI)					
	PD	Control		Crude^b^	*P*-value	Model 1^b,c^	*P*-value	Model 2^b,c,d^	*P*-value
Men (*n* = PD: 3919, Control: 15,676)									
Overall cancer (*n* = 1986)	16.83	20.22	−3.39 (−5.70 to −1.09)	0.83 (0.73–0.94)	0.003^a^	0.66 (0.58–0.75)	<0.001^a^	0.66 (0.58–0.75)	<0.001^a^
Gastric cancer (*n* = 419)	3.65	4.11	−0.47 (−1.50 to 0.56)	0.86 (0.66–1.13)	0.277	0.68 (0.52–0.89)	0.005^a^	0.68 (0.52–0.89)	0.005^a^
Thyroid cancer (*n* = 39)	0.28	0.38	−0.11 (−0.41 to 0.20)	0.64 (0.25–1.65)	0.359	0.50 (0.19–1.29)	0.152	0.51 (0.20–1.31)	0.159
Colorectal cancer (*n* = 390)	3.03	3.84	−0.81 (−1.79 to 0.17)	0.79 (0.59–1.05)	0.106	0.65 (0.48–0.86)	0.003^a^	0.65 (0.49–0.87)	0.004^a^
Lung cancer (*n* = 552)	4.30	5.39	−1.10 (−2.26 to 0.06)	0.80 (0.63–1.02)	0.074	0.68 (0.53–0.86)	0.002^a^	0.68 (0.53–0.87)	0.002^a^
Hepatic cancer (*n* = 259)	2.01	2.53	−0.52 (−1.31 to 0.27)	0.80 (0.56–1.14)	0.215	0.64 (0.45–0.92)	0.016^a^	0.65 (0.45–0.93)	0.019^a^
Bladder cancer (*n* = 153)	1.28	1.47	−0.19 (−0.80 to 0.42)	0.89 (0.57–1.39)	0.614	0.76 (0.48–1.19)	0.225	0.76 (0.48–1.18)	0.222
Pancreatic cancer (*n* = 104)	0.56	1.06	−0.50 (−1.00 to 0.00)	0.53 (0.28–1.03)	0.060	0.41 (0.21–0.80)	0.009^a^	0.41 (0.21–0.80)	0.009^a^
Gallbladder and biliary duct (*n* = 117)	1.17	1.08	0.08 (−0.45 to 0.62)	1.06 (0.66–1.71)	0.805	0.91 (0.56–1.47)	0.691	0.92 (0.57–1.50)	0.741
Kidney cancer (*n* = 60)	0.89	0.50	0.40 (0.02 to 0.78)	1.65 (0.93–2.92)	0.089	1.37 (0.76–2.45)	0.294	1.40 (0.78–2.50)	0.265
Hematological malignancy (*n* = 110)	0.95	1.05	−0.10 (−0.62 to 0.41)	0.87 (0.52–1.45)	0.586	0.73 (0.43–1.23)	0.233	0.73 (0.43–1.23)	0.230
Women (*n* = PD: 4462, Control: 17,848)									
Overall cancer (*n* = 1179)	7.65	9.30	−1.66 (−2.99 to −0.32)	0.81 (0.69–0.95)	0.011^a^	0.55 (0.46–0.64)	<0.001^a^	0.56 (0.48–0.66)	<0.001^a^
Gastric cancer (*n* = 270)	2.01	2.03	−0.02 (−0.66 to 0.61)	1.00 (0.73–1.36)	0.973	0.67 (0.49–0.93)	0.015^a^	0.69 (0.51–0.96)	0.025^a^
Thyroid cancer (*n* = 121)	0.79	0.93	−0.14 (−0.56 to 0.28)	0.80 (0.49–1.30)	0.367	0.62 (0.38–1.01)	0.055	0.65 (0.39–1.06)	0.085
Colorectal cancer (*n* = 247)	1.20	1.99	−0.78 (−1.38 to −0.18)	0.60 (0.41–0.88)	0.009^a^	0.42 (0.29–0.63)	<0.001^a^	0.43 (0.29–0.64)	<0.001^a^
Lung cancer (*n* = 204)	1.53	1.52	0.02 (−0.53 to 0.56)	1.03 (0.72–1.47)	0.867	0.71 (0.49–1.02)	0.064	0.75 (0.52–1.08)	0.119
Hepatic cancer (*n* = 105)	0.70	0.80	−0.09 (−0.48 to 0.29)	0.89 (0.53–1.49)	0.646	0.59 (0.35–1.02)	0.057	0.59 (0.34–1.02)	0.058
Bladder cancer (*n* = 41)	0.25	0.32	−0.07 (−0.31 to 0.17)	0.76 (0.32–1.82)	0.542	0.54 (0.22–1.29)	0.164	0.54 (0.22–1.29)	0.164
Pancreatic cancer (*n* = 97)	0.37	0.80	−0.43 (−0.80 to −0.05)	0.46 (0.23–0.92)	0.027^a^	0.31 (0.16–0.62)	0.001^a^	0.32 (0.16–0.63)	0.001^a^
Gallbladder and biliary duct (*n* = 84)	0.66	0.62	0.05 (−0.30 to 0.39)	1.07 (0.62–1.85)	0.809	0.77 (0.44–1.34)	0.351	0.79 (0.45–1.38)	0.399
Kidney cancer (*n* = 32)	0.25	0.24	0.01 (−0.20 to 0.23)	1.02 (0.42–2.47)	0.974	0.78 (0.32–1.92)	0.587	0.72 (0.29–1.80)	0.484
Hematological malignancy (*n* = 65)	0.25	0.53	−0.29 (−0.59 to 0.02)	0.45 (0.19–1.04)	0.062	0.32 (0.14–0.75)	0.009^a^	0.32 (0.14–0.75)	0.009^a^

*CI* confidence interval, *PD* Parkinson’s disease.

^a^Stratified Cox proportional hazard regression model, Significance at *P* < 0.05.

^b^Stratified by age, sex, income, and region of residence.

^c^Adjusted for obesity, smoking, alcohol consumption, and Charlson Comorbidity Index scores.

^d^Adjusted for systolic blood pressure, diastolic blood pressure, fasting blood glucose, and total cholesterol.

In the age-stratified subgroup analyses, only the subgroup of patients with PD aged ≥60 years exhibited associations similar to those observed in the main analysis, with inverse associations between PD and the development of overall, gastric, thyroid, lung, hepatic, and pancreatic cancer and hematological malignancy (Table 4).

**Table 4:** Age-stratified subgroup analysis of crude and adjusted hazard ratios of Parkinson’s disease for various cancers.

	Incident rate per 1000 person-year		Incident rate difference per 1000 person-years (95% CI)	Hazard ratio for cancer (95% CI)					
	PD	Control		Crude^b^	*P*-value	Model 1^b,c^	*P*-value	Model 2^b,c,d^	*P*-value
Age < 60 (*n* = PD: 313, Control: 1252)									
Overall cancer (*n* = 78)	4.67	5.08	−0.41 (−3.22 to 2.41)	0.91 (0.51–1.62)	0.746	0.89 (0.48–1.64)	0.701	0.87 (0.47–1.62)	0.668
Gastric cancer (*n* = 17)	1.64	0.94	0.71 (−0.59 to 2.00)	2.05 (0.71–5.92)	0.187	2.23 (0.69–7.27)	0.182	2.95 (0.71–12.17)	0.136
Thyroid cancer (*n* = 14)	0.66	0.93	−0.28 (−1.45 to 0.89)	0.67 (0.15–3.00)	0.602	0.65 (0.14–3.02)	0.578	0.64 (0.13–3.16)	0.583
Colorectal cancer (*n* = 17)	0.65	1.17	−0.51 (−1.80 to 0.78)	0.58 (0.13–2.67)	0.488	0.55 (0.12–2.55)	0.444	1.08 (0.23–5.08)	0.925
Lung cancer (*n* = 10)	0.65	0.62	0.03 (−0.95 to 1.02)	1.08 (0.23–5.08)	0.925	1.13 (0.19–6.75)	0.897	1.28 (0.18–9.16)	0.808
Hepatic cancer (*n* = 9)	0.00	0.70	−0.70 (−1.63 to 0.24)	N/A	0.995	N/A	0.997	N/A	0.997
Bladder cancer (*n* = 2)	0.00	0.15	−0.15 (−0.60 to 0.29)	N/A	0.997	N/A	1.000	N/A	1.000
Pancreatic cancer (*n* = 3)	0.00	0.23	−0.23 (−0.77 to 0.31)	N/A	0.997	N/A	1.000	N/A	1.000
Gallbladder and biliary duct (*n* = 3)	0.00	0.23	−0.23 (−0.77 to 0.31)	N/A	0.997	N/A	1.000	N/A	1.000
Kidney cancer (*n* = 4)	0.33	0.23	0.09 (−0.53 to 0.72)	1.24 (0.13–11.93)	0.855	N/A	1.000	N/A	1.000
Hematological malignancy (*n* = 7)	0.65	0.39	0.27 (−0.56 to 1.09)	1.63 (0.32–8.40)	0.561	1.16 (0.14–9.60)	0.894	1.15 (0.13–10.21)	0.898
Age ≥ 60 (*n* = PD: 8068, Control: 32,272)									
Overall cancer (*n* = 3087)	12.06	14.73	−2.66 (−3.99 to −1.34)	0.82 (0.74–0.90)	<0.001^a^	0.61 (0.55–0.68)	<0.001^a^	0.62 (0.56–0.69)	<0.001^a^
Gastric cancer (*n* = 672)	2.79	3.09	−0.31 (−0.91 to 0.30)	0.89 (0.73–1.10)	0.277	0.66 (0.54–0.82)	<0.001^a^	0.67 (0.54–0.82)	<0.001^a^
Thyroid cancer (*n* = 146)	0.56	0.67	−0.10 (−0.38 to 0.17)	0.77 (0.49–1.21)	0.257	0.58 (0.37–0.92)	0.019^a^	0.60 (0.38–0.95)	0.028^a^
Colorectal cancer (*n* = 620)	2.09	2.93	−0.84 (−1.42 to −0.26)	0.55 (0.43–0.69)	<0.001^a^	0.55 (0.44–0.70)	<0.001^a^	0.86 (0.70–1.05)	0.147
Lung cancer (*n* = 746)	2.87	3.42	−0.55 (−1.18 to 0.08)	0.86 (0.70–1.05)	0.147	0.69 (0.56–0.85)	<0.001^a^	0.70 (0.57–0.86)	0.001^a^
Hepatic cancer (*n* = 355)	1.36	1.63	−0.27 (−0.70 to 0.16)	0.85 (0.64–1.14)	0.283	0.66 (0.49–0.89)	0.006^a^	0.66 (0.49–0.89)	0.007^a^
Bladder cancer (*n* = 192)	0.74	0.88	−0.14 (−0.45 to 0.18)	0.88 (0.59–1.30)	0.508	0.72 (0.48–1.07)	0.102	0.72 (0.48–1.07)	0.103
Pancreatic cancer (*n* = 198)	0.49	0.96	−0.48 (−0.80 to −0.15)	0.51 (0.32–0.82)	0.005^a^	0.37 (0.23–0.59)	<0.001^a^	0.38 (0.23–0.61)	<0.001^a^
Gallbladder and biliary duct (*n* = 88)	0.95	0.87	0.08 (−0.24 to 0.40)	1.09 (0.76–1.56)	0.650	0.87 (0.60–1.25)	0.435	0.89 (0.61–1.28)	0.512
Kidney cancer (*n* = 168)	0.54	0.36	0.18 (−0.04 to 0.39)	1.42 (0.87–2.32)	0.163	1.13 (0.68–1.86)	0.640	1.13 (0.69–1.87)	0.625
Hematological malignancy (*n* = 531)	0.54	0.79	−0.25 (−0.55 to 0.04)	0.66 (0.42–1.05)	0.078	0.53 (0.34–0.85)	0.008^a^	0.53 (0.34–0.85)	0.008^a^

*CI* confidence interval, *PD* Parkinson’s disease.

^a^Stratified Cox proportional hazard regression model, Significance at *P* < 0.05.

^b^Stratified by age, sex, income, and region of residence.

^c^Adjusted for obesity, smoking, alcohol consumption, and Charlson Comorbidity Index scores.

^d^Adjusted for systolic blood pressure, diastolic blood pressure, fasting blood glucose, and total cholesterol.

## Discussion

This nationwide, longitudinal study revealed that the probability of subsequent overall cancer development in patients with PD was 0.63 times that in the matched control sample. Furthermore, we discovered that the probabilities of development of gastric, thyroid, colorectal, lung, hepatic, and pancreatic cancer and hematological malignancy were significantly lower in the PD group than in the control group.

Our results of an inverse association between PD and overall cancer and many different types of cancer are consistent with the majority of prior studies’ findings^3–5,9–11^, but not all^12^. In a study of the risk of cancer development among 11,7863 patients with PD in Sweden, the risk of smoking-related cancers was lower than in participants without PD (HR = 0.87, 95% CI = 0.79–0.96)^13^. One study in a Danish population revealed a decreased risk for lung (standardized incidence ratio [SIR] = 0.38, 95% CI = 0.3–0.5), laryngeal (SIR = 0.47, 95% CI = 0.2–1.1), and urinary bladder (SIR = 0.52, 95% CI = 0.4–0.7) cancer and an increased risk for melanoma/skin cancer (SIR = 1.95, 95% CI = 1.4–2.6) and breast cancer (SIR = 1.24, 95% CI = 1.0–1.5) in 14,088 patients with PD compared with participants without PD^14^. In a British study of 219,194 patients with PD, lower risks for the development of any cancer (standardized rate ratio = 0.95, 95% CI = 0.91–0.93) and colon cancer were observed compared with participants without PD, as well as a higher risk for breast cancer and melanoma^4^. Our discovery of lower probabilities for the development of cancers in patients with PD corroborates the results of European studies (both for cancers associated with and those not known to be associated with smoking), and that of higher risks of melanoma and breast cancer was corroborated in American studies^15,16^.

This general inverse association was also demonstrated in a meta-analysis of 29 studies (107,598 patients with PD): the aggregate risk for cancer in patients with PD compared with controls was 0.73 (95% CI = 0.63–0.83), and that after excluding skin tumors was 0.69 (95% CI = 0.62–0.78)^17^. In a meta-analysis of 50 observational studies by Catala-Lopez et al., the estimated reduction in malignant neoplasm-related morbidity among patients with PD was 17%, and reductions were especially observed for lung, bladder, bowel, prostate, and uterus cancers and blood malignancies^18^. A recent meta-analysis, with qualitative synthesis of 23 studies, revealed that patients with PD had a lower overall cancer risk (relative risk = 0.94, 95% CI = 0.93–0.95) and lower risk of digestive system, lung, and urinary system cancers^19^. Hence, a large number of nationwide population-based cohort studies and meta-analyses have provided robust epidemiological evidence for the inverse relationship between PD and cancer.

Although this was an observational study that did not address to pathophysiological mechanisms, the observed epidemiological links between PD and cancer have several plausible explanations. First, strong environmental and lifestyle factors influence the altered susceptibility to cancer in patients with PD, especially smoking^20,21^. Smoking plays a protective role against the development of PD^22^, whereas it is directly associated with certain cancer types. This explanation is supported by a meta-analysis in which the decreased risk of cancer in patients with PD was particularly the case for smoking-related cancers (40% risk reduction)^17^. However, our analysis revealed that, among smoking-related cancers, significant inverse relations with PD were identified for gastric, lung, hepatic, and pancreatic cancer but not for bladder or kidney cancer. Furthermore, certain types of cancer that are not related to smoking may also have a close, inverse association with PD. Therefore, genetic factors likely also lower or raise the susceptibility of patients with PD to malignant neoplasms. From such a genetic perspective, the association and interplay between cancer and PD may be due to shared biological processes, such as mitochondrial dysfunction, oxidative stress, DNA damage, cell cycle abnormalities, inflammatory factors, and impaired mitophagy (pathways involved in mitochondrial quality control)^23^. Transcriptomic association studies have identified combined deregulation of the aforementioned biological processes in both cancer and neurodegenerative diseases, suggesting that shared alterations in biological processes may play a role in the association of PD and cancer^24–26^. Third, the most compelling explanations for associations between PD and certain cancer types are communal genetic mutations occurring in both diseases^27^. Proteins coded by genes implicated in PD pathogenesis are involved in various cellular pathways, and mutations in these genes affect both neurodegenerative and tumorigenic processes. Specifically, α-synuclein, encoded by *PARK1*, the major protein component of Lewy bodies in the brains of patients with PD, may be involved in certain neoplastic formations, and its downregulation has been observed in the other cancer types^28,29^. Dysfunction of parkin, encoded by *PARK2*, may lead to the growth of certain cancer types and the suppression of others^30,31^. Increased expression of DJ-1, encoded by *PARK7*, has been observed in melanomas and breast, lung, colorectal, uterine, hepatocellular, and nasopharyngeal cancers^32,33^. *PARK6* (PTEN-induced kinase-1), *PARK8* (leucine rich repeat kinase 2), and other proteins also play a role in both diseases^34–36^. Despite accumulating evidence based on genetic, cellular, and molecular biology studies of cancer and neurodegeneration, the molecular underpinnings of the link between these two disorders remain unclear. Further investigation is necessary to close the remaining knowledge gaps.

To date, most of the epidemiological studies pertaining to the association between PD and cancer, as well as the latest meta-analysis, were performed in white participants. However, genetic mutations differ across ethnicities, as seen in the disparities in the risk and prevalence of cancer between Eastern and Western populations. However, we are aware of three studies examining the link between PD and cancer in Asians^6–8^, only two of which were large-scale, nationwide surveys have been conducted in the past decade, one in Taiwan and the other in Korea. Western patients with PD are most commonly reported to have decreased risks of stomach, colorectal, lung, blood, and bladder cancer, as well as a decrease in the overall cancer risk. Those discoveries are consistent with those of the Korean study by Park et al.^8^, but contrary to those of the Taiwanese study by Lin et al.^6^. In the latter, the 16 of the 19 types of cancer investigated had a higher HR in patients with PD, and the overall risk of cancer was 1.58-fold higher^6^. Park et al. believed that the inconsistencies in the results of the two Asian studies may be attributed to the differences in the diagnostic accuracy of PD^8^. Intriguingly, however, our analyses, in which claim codes were used as they were in the Taiwanese study, yielded contrasting results to those in the Taiwanese study, but similar to those in the Korean study, which complicates the apparent association of PD and cancer. We speculated that, over and above genetic backgrounds, various environmental factors, such as diet, lifestyle, physical activity and exercise, alcohol consumption, smoking status, exposure to toxins or pesticides, and medication use, may result in these discordant results among Asian populations.

Moreover, the comparison point between our study and another Korean study^8^ is that the study population and the confounding factors adjusted for analyses are different. A previous Korean study published in 2019 used all relevant data accumulated in the National Health Insurance Service (NHIS) database, whereas the present study used data from the Korean NHIS-Health Screening Cohort, a sample of participants aged 40–79 years who received medical checkups between 2002 and 2003. This cohort included information on risk factors obtained from questionnaires and bioclinical laboratory results. Overall, our study had fewer patients with PD than a previous study, but we had a control group with demographic and socioeconomic information (age, sex, income, and residential area) that matched that of the patient group. We also adjusted for the comorbidity index, in addition to well-known risk factors such as alcohol consumption and smoking, which the previous Korean study did not control for.

The inverse association with overall cancer and most cancer types in patients with PD in this study were relatively consistent with results in the subgroup analyses based on patient sex. The only exception was observed with kidney cancer, but the result was not statistically significant. In contrast, age slightly affected the inverse association of PD and cancer. No changes were observed for participants aged ≥60 years, whereas the HRs of gastric, colorectal, and lung cancer were not statistically significant in participants aged <60 years. As with differences between countries, environmental factors may also explain these intergenerational differences.

Several caveats of our study must be acknowledged. First, the ascertainment of PD was determined using the International Classification of Diseases, 10th edition (ICD-10) codes G20 (Parkinson’s disease), and only patients who visited the clinic more than two times with that code were included in the PD group. However, previous studies defined patients who had newly diagnosed PD as patients who were assigned both the ICD-10 codes (G20) and rare intractable disease (RID) registration code (V124). Unlike these studies, our study did not consider this RID registration code, which is given after qualified physicians evaluate whether the diseases meet the diagnostic criteria for each RID, and only categorized participants as having PD if they had multiple claim codes of the corresponding ICD-10. Consequently, the number of patients with PD in the present study may have been either underestimated or overestimated. Nonetheless, our results are consistent with the results of most previous studies that investigated the association between PD and cancer, including a large-scale study in Korea. Second, we cannot rule out the effects of possible unmeasured confounders, such as coffee intake, physical activity, family history of malignancy, and genetic factors, on the associations between PD and cancer. Third, the potential role of medication use, especially of anti-parkinsonian drugs, may act as a confounding factor, but was not considered in the data analyses. Further investigation of the effect of PD on the cancer risk, controlled for a variety of additional potential confounders, is warranted. Fourth, the increase in visits to health care institutions because of the diagnosis of PD is likely to act as a surveillance bias in interpreting the significant association between PD and cancer occurrence. Furthermore, we found a lower incidence of cancer in patients with PD, which may have led to underdiagnosis of cancer in the PD group because non-motor symptoms of PD, such as fatigue, weight loss, and nausea, can obscure the initial symptoms of cancer. Fifth, pathological changes in PD may begin in the prodromal phase before the onset of classic signs of PD, which may be associated with cancer development. However, estimates of the duration of the prodromal phase range from months to decades, and the features also vary broadly. Thus, it is not easy to clearly define the prodromal phase and determine the association between it and cancer. Moreover, we were unable to identify prodromal PD cases using the data for this study because we identified PD cases and controls from only the claims data without access to clinically detailed information. Although we did not confirm the start date precursor timing in the PD patient group, we defined the index date as the PD clinical diagnosis data and assigned the same index date to the matched control group. Therefore, the conditions for pathological changes in PD, which may be associated with the occurrence of cancer, are equivalent in both groups.

Notwithstanding the abovementioned limitations, our study has notable strengths. We analyzed nationwide claims data, allowing all participants to be accurately traced and reducing the loss to follow-up. In addition, the control participants were matched to patients with PD according to the most important confounding factors, including not only age and sex but also income and residential area. Finally, we adjusted extensively for PD- and cancer-related comorbidities and covariates, such as obesity, smoking, alcohol consumption, comorbidity scores, blood pressure, and blood and cholesterol levels, and we performed subgroup analyses to assess the robustness of the main findings. In particular, although smoking status has a pivotal effect on the relationship between the two diseases, no adjustment for smoking history was made in the statistical models of most prior studies. Thus, our study provides sufficient additional support for the relationship between PD and cancer, a matter of some controversy in the literature.

In our investigation, patients with PD had lower probabilities of subsequent cancer and different types of cancer than did participants without PD. Further research is warranted to replicate our findings and to clearly explain the biological mechanisms of the apparent inverse association between PD and cancer.

## Methods

### Data sources and ethical considerations

Participants in this study were selected from the Korean NHIS-Health Screening Cohort database, which includes details regarding health insurance claim codes (for procedures and prescriptions); diagnostic codes, according to the ICD-10; death records; socioeconomic data; and health check-up data. To construct the NHIS-Health Screening Cohort database, a sample cohort was selected from the 2002 and 2003 health screening participants, who were aged between 40 and 79 in 2002 and followed up through 2013. This cohort included 514,866 health screening participants who comprised a random selection of 10% of all health screening participants in 2002 and 2003^37^.

This study was approved by the ethics committee of Hallym University (2019-10-023), and the requirement for written informed consent was waived by the Institutional Review Board because this was a recording-based study with no patient contact. We conducted all analyses in accordance with the guidelines and regulations of the ethics committee of Hallym University.

### Study population and participant selection

We initially selected participants newly diagnosed with PD from January 1, 2002 to December 31, 2019 (*n* = 9437), from a total of 514,866 participants with 895,300,177 medical claim codes. The diagnosis of PD was determined using the ICD-10 code G20 (Parkinson’s disease), and only patients who visited the clinic ≥2 times with that code were included in the PD group. Moreover, we excluded patients who had first been diagnosed with PD in 2002 from the analyses (washout period, *n* = 404).

The remaining individuals in the database (those who were not included in the PD group, *n* = 505,429 were selected as control participants, but excluded those who had been diagnosed with PD once (*n* = 3554).

Patients in the PD group were randomly matched to control participants at a 1:4 ratio according to age, sex, income, and residential area. The index date was defined as the date of the first PD diagnosis. Control participants were assigned the same index date as the patients with which they were matched. Accordingly, participants who died before the index date and those who had a history of cancer before the index date were excluded from each group. Hence, 652 patients and 468,351 control participants were ruled out during the matching process (Fig. 1).

### Covariates

Relevant covariates of this study included age, sex, income, region of residence, obesity, smoking, alcohol consumption, systolic blood pressure (SBP, mmHg), diastolic blood pressure (DBP, mmHg), fasting blood glucose (mg/dL), total cholesterol (mg/dL), and CCI.

Participants were allocated to 10 age groups, with 5-year intervals, from 40-44 to 85 years old and older. Participants were stratified into income classes, from class 1 (lowest income) to class 5 (highest income). The region of residence was defined as urban (Seoul, Busan, Daegu, Incheon, Gwangju, Daejeon, and Ulsan) or rural (Gyeonggi, Gangwon, Chungcheongbuk, Chungcheongnam, Jeollabuk, Jeollanam, Gyeongsangbuk, Gyeongsangnam, and Jeju).

Participants’ smoking status were defined as nonsmoker, past smoker, and current smoker. Alcohol consumption was classified according to frequency: <1 time a week and ≥1 time a week. Obesity was determined using the body mass index (kg/m^2^) with the following categories: <18.5 (underweight), ≥18.5 to <23 (normal), ≥23 to <25 (overweight), ≥25 to <30 (obese I), and ≥30 (obese II), as stated in the Asia-Pacific criteria^38^. The CCI, which is used to assess 17 comorbidities, ranges from a score of 0 (no comorbidities) to 29 (multiple comorbidities)^39^. The CCI in this study was calculated for 15 comorbidities, excluding cancer and metastatic cancer.

### Cancer ascertainment

During the follow-up period, we identified the following 10 site-specific cancers: gastric cancer (ICD-10 code: C16), thyroid cancer (C73), colorectal cancer (C18 to C21 and D010 to D013), lung cancer (C34 and D022), hepatic cancer (C22 and D015), bladder cancer (C67 and D090), pancreatic cancer (C25 and D017), gallbladder and biliary duct cancer (C23 and C24), kidney cancer (C64), and hematological malignancy (C81 to C96). We included only participants who were diagnosed with the same ICD-10 code at least twice to select patients with severe cancer.

### Statistical analyses

Descriptive statics are presented using frequencies and proportions for categorical variables and means with standard deviations for continuous variables. Propensity-score overlap weighting was performed for exact matching of participants according to covariates and to determine the effective sample size. The propensity score was calculated via multivariable logistic regression with all relevant covariates. Overlap weighting was calculated with a range of 0 and 1; participants in the PD group were weighted according to the probability of the propensity score, and control participants were weighted according to the probability of one minus the propensity score. The standardized difference was used to compare the demographic and clinical characteristics between the PD and control groups before and after weighting. An absolute standardized difference of <0.20 represented a good balance between the groups for an included variable.

Propensity-score overlap-weighted Cox proportional-hazards regression analysis was used to estimate HRs and 95% CIs for overall cancer and each of 10 different cancers among patients with PD compared with control participants. In addition to the unadjusted model (crude model), multivariable Cox proportional-hazards analysis was conducted to adjust for different potential confounders measured at baseline. We controlled for the covariates of obesity, smoking status, alcohol consumption, and CCI scores in model 1 and additionally adjusted for SBP, DBP, fasting blood glucose, and total cholesterol in model 2. Time to development of overall and each of 10 cancers in participants with and those without PD was calculated using Kaplan–Meier curves and compared using the log-rank test.

We conducted subgroup analyses with respect to age (<60 years old vs ≥60 years old) and sex (men vs. women), using stratified Cox proportional-hazards models.

A two-tailed *P*-value < 0.05 was deemed significant. All statistical analyses were conducted with SAS version 9.4 (SAS Institute Inc., Cary, NC, USA).

## Data Availability

All data are available from the database of National Health Insurance Sharing Service (NHISS) https://nhiss.nhis.or.kr. NHISS allows access to all of this data for the any researcher who promises to follow the research ethics at some cost. If you want to access the data of this study, you can download it from the website after promising to follow the research ethics.
